# Correlation risk analysis of serum inflammatory factors and 25-hydroxyvitamin D in vascular lesions in type 2 diabetes patients

**DOI:** 10.5937/jomb0-59322

**Published:** 2026-01-28

**Authors:** Qian Zhang, Yuyan Zhu, Hui Li, Xue Wu, Cheng Wu, Guang Li, Chao Yang

**Affiliations:** 1 Jinan Third People's Hospital, Department of Endocrinology and Metabolism, Jinan City, China; 2 Huai'an Clinical Medical College of Jiangsu University, Department of Operating Room, Huai'an City, China; 3 Zibo Central Hospital, Department of Anesthesiology and Surgery, Zibo City, China; 4 Zhongshan Hospital Affiliated to Fudan University, Endocrinology Department, Shanghai, China; 5 The Second People's Hospital of Liaocheng, Department of Endocrinology and Metabolism, Linqing City, China

**Keywords:** correlation risk analysis, serum inflammatory factor, 25-hydroxyvitamin D, vascular lesions, type 2 diabetes, analiza rizika korelacije, serumski inflamatorni faktor, 25-hidroksivitamin D, vaskularne lezije, dijabetes tipa 2

## Abstract

**Background:**

To explore serum inflammatory factors and 25-hydroxyvitamin D and diabetic vascular lesions in patients with T2DM.

**Methods:**

A total of 362 adult (aged 18 years) patients with T2DM hospitalised from April 2022 to June 2024 were selected as research subjects. A patient's body mass index (BMI), systolic blood pressure (SBP), diastolic blood pressure (DBP), age, sex, and length of illness were among the general data gathered. Blood parameters, glycated haemoglobin (HbA1c), fasting blood glucose (FPG), total cholesterol (TC), triglycerides (TG), low-density lipoprotein cholesterol (LDL-C), inflammatory factors [C-reactive protein (CRP), interleukin-6 (IL-6)], and 25-hydroxyvitamin D [25(OH) D] were determined. The homeostatic model assessment of insulin resistance index (HOMA-IR) and the systemic inflammatory index (SII) were computed. The vascular lesions were divided into three groups: the simple T2DM group (155 patients), the T2DM microvascular lesion group (122 patients), and the T2DM macrovascular lesion group (85 patients). One-way analysis of variance, the Kruskal-Wallis H test, or the c2 test was used for comparisons among multiple groups. The relationship between 25(OH) D and several inflammatory markers was examined using Spearman correlation analysis, and the factors influencing diabetic vascular lesions were analysed using multivariate logistic regression analysis.

**Results:**

The levels of 25(OH) D in both the T2DM microvascular lesion group and the T2DM macrovascular lesion group were significantly lower than those in the simple T2DM group (P<0.05). The level of IL-6 in the T2DM microvascular lesion group was significantly greater than that in the simple T2DM group (P<0.05). Compared to the simple T2DM group and the T2DM microvascular lesion group, the T2DM macrovascular lesion group had significantly higher levels of CRP IL-6, and the SII (P< 0.05). Multivariate logistic regression analysis results showed that after controlling for confounding variables. With the simple T2DM group as the reference, CRP (OR= 5.35, 95% CI 1.49-19.13) and 25(OH) D (OR= 0.88, 95% CI 0.78-0.98) were the influencing factors of microvascular lesions in T2DM patients; CRP IL-6, SII, and 25(OH) D were influencing factors of macrovascular lesions in type 2 diabetes mellitus (T2DM) patients (OR = 14.99, 95% CI 2.84-79.13; OR= 27.92, 95% CI 4.24-183.92; OR= 1.01, 95% CI 1.00-1.02; OR= 0.74, 95% CI 0.60-0.92). Spearman correlation analysis revealed a negative correlation between CRP and IL-6 (r= 0.600, P<0.001) and between CRP and the SII (r= 0.256, P<0.001), while IL-6 showed a positive correlation with the SII (r= 0.307, P&lt; 0.001). Additionally, 25(OH) D had a negative correlation with CRP (r=-0.102, P= 0.052), IL-6 (r=-0.115, P= 0.028), and the SII (r=-0.141, P=0.007).

**Conclusions:**

Compared with those in patients with T2DM microvascular lesions, the levels of the inflammatory factors CRP and IL-6 and the SII in the serum of patients with T2DM macrovascular lesions are greater, and the level of 25(OH) D is lower.

## Abstract (sr)

**Uvod:**

Cilj je bio da se ispitaju serumski inflamatorni faktori, 25-hidroksivitamin D i vaskularne lezije kod pacijenata sa dijabetesom tipa 2 (T2DM).

**Metode:**

U studiju su uključena ukupno 362 odrasla pacijenta (uzrasta 18 godina) sa T2DM, hospitalizovana u periodu od aprila 2022. do juna 2024. godine. Prikupljeni su opšti podaci kao što su indeks telesne mase (BMI), sistolni krvni pritisak (SBP), dijastolni krvni pritisak (DBP), starost, pol i trajanje bolesti. Određeni su krvni parametri, glikozilisan hemoglobin (HbA1c), nivo glukoze u krvi na prazan stomak (FPG), ukupni holesterol (TC), trigliceridi (TG), holesterol lipoproteina niske gustine (LDL-C), inflamatorni faktori [C-reaktivni protein (CRP), interleukin-6 (IL-6)] i 25-hidroksivitamin D [25(OH) D]. Indeks insulinske rezistencije je izračunat po modelu homeostaze (HOMA-IR) kao i sistemski inflamatorni indeks (SII). Pacijenti su podeljeni u tri grupe prema tipu vaskularnih lezija: grupa sa prostim T2DM-om (155 pacijenata), grupa sa mikro-vaskularnim lezijama (122 pacijenta) i grupa sa makro-vaskularnim lezijama (85 pacijenata). Za poređenje između više grupa korišćeni su jednofaktorska analiza varijanse, Kruskal-Wallis H test ili hi-kvadrat (c2) test. Povezanost između 25(OH) D i više inflamatornih markera je ispitana Spirmanovom korelacionom analizom, a faktori koji utiču na vaskularne lezije kod dijabetesa su analizirani višestrukom logističkom regresijom.

**Rezultati:**

Nivoi 25(OH) D u grupama sa mikroi makro-vaskularnim lezijama kod T2DM pacijenata bili su značajno niži u poređenju sa grupom sa prostim T2DM-om (P&lt; 0,05). Nivo IL-6 u grupi sa mikrovaskularnim lezijama bio je značajno viši nego u grupi sa prostim T2DM-om (P&lt; 0,05). U poređenju sa prostim T2DM-om i mikro-vaskularnom grupom, grupa sa makrovaskularnim lezijama imala je značajno više vrednosti CRP-a, IL-6 i SII (P&lt; 0,05). Rezultati višestruke logističke regresione analize pokazali su da su, nakon kontrole za konfuzione varijable, CRP (OR= 5,35, 95% CI 1,49-19,13) i 25(OH) D (OR= 0,88, 95% CI 0,78-0,98) faktori koji utiču na mikrovaskularne lezije kod T2DM pacijenata; CRP IL-6, SII i 25(OH) D su faktori koji utiču na makrovaskularne lezije kod T2DM pacijenata (OR= 14,99, 95% CI 2,84-79,13; OR= 27,92, 95% CI 4,24-183,92; OR= 1,01, 95% CI 1,00-1,02; OR= 0,74, 95% CI 0,60-0,92). Spirmanova korelaciona analiza pokazala je negativnu korelaciju između CRP-a i IL-6 (r= 0,600, P&lt; 0,001) kao i između CRP-a i SII (r= 0,256, P&lt; 0,001), dok je IL-6 bio pozitivno korelisan sa SII (r= 0,307, P&lt; 0,001). Takođe, 25(OH) D je bio negativno korelisan sa CRP-om (r=-0,102, P= 0,052), IL-6 (r=-0,115, P= 0,028) i SII (r=-0,141, P=0,007).

**Zaključak:**

U poređenju sa pacijentima sa mikrovaskularnim lezijama, pacijenti sa makrovaskularnim lezijama kod T2DM imaju više nivoe inflamatornih faktora CRP i IL-6 i viši sistemski inflamatorni indeks (SII), dok je nivo 25(OH) D niži.

## Introduction

Continuous blood glucose monitoring and effective control are needed to alleviate its social burden [1] [2]. Diabetes not only easily causes microvascular damage but can also lead to macrovascular lesions such as coronary heart disease, cerebral infarction and atherosclerotic lesions of the lower limbs (LEADs) [3]. A 3B study in China revealed that among outpatients with T2DM, 14.6% had cardiovascular disease (CVD), and 10.1% had cerebrovascular disease[4]. According to data released by the World Health Organisation in 2022, 17.9 million people die from cardiovascular disease (CVD) each year, accounting for one in every three deaths globally. The classic inflammatory pathway involving IL-1, IL-6, and hyper sensitive C-reactive protein (hs-CRP) accelerates the onset and progression of vascular atherosclerosis [5] [6] [7] [8]. However, the differences in these inflammatory factors between macrovascular and microvascular diabetic lesions remain unclear.

Therefore, our study provides a theoretical basis for early and precise intervention, as well as effective prevention and treatment of diabetic vascular complications, by comparing the differences in inflammatory indicators between macrovascular and microvascular lesions in patients with diabetes.

## Materials and methods

### Research subjects

Three hundred sixty-two adult patients (aged 18 years or older) with type 2 diabetes who were admitted between April 2022 and June 2024 were selected as research participants; all of them met the World Health Organisation's 2022 diabetes diagnostic and categorisation standards.

Exclusion criteria: (1) other types of diabetes other than T2DM; (2) acute complications of diabetes occurring within one month; and (3) history of severe liver and kidney dysfunction, infection, tumours, or autoimmune diseases.

### Collection of general information

Patient age, sex, duration of diabetes, height, weight, systolic blood pressure (SBP), diastolic blood pressure (DBP), smoking history, drinking history, and previous medical history were recorded, and the body mass index (BMI) was calculated.

### Measurement of laboratory indicators

(1) In order to identify the following signs, fasting venous blood was drawn early the next day after all research participants had fasted for eight to twelve hours. The Johnson & Johnson VITROS 5600 fully automatic biochemical and immunoassay analyser was used to test the following parameters: fasting plasma glucose (FPG), total cholesterol (TC), triglycerides (TG), low-density lipoprotein cholesterol (LDL-C), and high-density lipoprotein cholesterol (HDL-C). (2) Routine blood analysis: Sysmex XN2800 was used as a fully automatic analyser. (3) Fasting serum C-peptide (FC-P), 25(OH) D, and iL-6 were detected with a Roche Cobas e801 electrochemiluminescence immunoassay analyzer. (4) Glycated haemoglobin A 1c (HbA 1c): This parameter is detected via high-performance liquid chromatography. (5) CRP: CRP was detected by the Pumen PA-980 fully automatic specific protein analyser. Currently, there is no unified standard for the critical value of the SII. It is determined using various methods, including previous studies, the quantile method, and the receiver operating characteristic curve.

### Diagnostic criteria and definitions

(1) Diagnostic basis for T2DM combined with microvascular lesions: Fundus examination (microhemangioma, haemorrhage, exudation, etc.). One or more positive signs (pain sensation, temperature sensation, vibration sensation, pressure sensation, and the ankle reflex), at least one positive symptom (numbness, pain, abnormal sensation, etc.), or two or more positive signs confirm the presence of microvascular lesions.

(2) Diagnostic basis for T2DM combined with macrovascular lesions: Coronary heart disease and/or cerebral infarction can occur after the patient is diagnosed with diabetes. Alternatively, coronary heart disease and/or cerebral infarction and/or LEAD was confirmed by coronary angiography/CT angiography, cranial CT/magnetic resonance imaging, or peripheral vascular colour ultrasound examination [quadripleural Doppler measured the patient's resting ankle/brachial index (ABI), and LEAD was diagnosed when the ABI was 0.90].

(3) Smoking history: A smoking history was defined as smoking at least one cigarette per day for a minimum of six months in a row or cumulatively.

(4) History of alcohol consumption: Within the past 12 months, regardless of the type of alcohol, as long as one has consumed alcohol at least once a month on average, they are considered to have a his tory of alcohol consumption.

### Grouping criteria

The vascular lesions were divided into three groups: the simple T2DM group, the T2DM microvascular lesion group, and the T2DM macrovascular lesion group. Carotid colour ultrasound did not reveal obvious intima-media thickening, multiple plaque formation, or arterial stenosis in the simple T2DM group or the T2DM microvascular lesion group.

### Statistical analysis

Utilising SPSS 25.0 software, the statistical analysis was performed. The data were normally distributed, represented by x̄ ± s values, and the groups were compared using one-way analysis of variance. The Kruskal-Wallis H test was employed for comparisons across several groups, and the nonnormally distributed data are represented as M (Q1, Q3). The χ^2 ^test was performed to compare groups, and count statistics are presented as cases (%). An analysis of the relationships between 25(OH) D and several inflammatory markers was conducted using Spearman correlation analysis. After controlling for associated confounding variables, multivariate logistic regression analysis was used to examine the factors influencing vascular lesions in individuals with type 2 diabetes mellitus (T2DM). P values less than 0.05 were regarded as statistically significant.

## Results

### Analysis of general clinical data

Among the 362 patients with T2DM, 231 were male (63.8%), and 131 were female (36.2%), with an average age of 57.5±11.7 years and an average duration of diabetes of 8.0 (2.0, 11.3) years. Among them, there were 155 patients (43%) in the simple T2DM group, 122 patients (34%) in the T2DM microvascular lesion group, and 85 patients (23%) in the T2DM macrovascular lesion group. Compared with the simple T2DM group, both the T2DM microvascular lesion group and the T2DM macro vascular lesion group were older, had longer dia betes durations, and higher SBP and FPG levels. The DBP was greater in the T2DM macrovascular lesion group, whereas the FC-P and HOMA-IR were lower.

Compared with the T2DM microvascular lesion group, the T2DM macrovascular lesion group was older, had a longer disease course, higher SBP and DBF, and lower FC-P and HOMA-IR (P< 0.05, Table 1).

**Table 1:** Comparison of baseline data of patients with T2DM in three groups.

Observation index	Simple T2DM group (155 cases)	T2DM microvascular lesion group (122 cases)	T2DM macrovascular lesion group (85 cases)	xIFIH value	P value
Gender [Example (%)]	104 (67.1)	81 (66.4)	46 (54.1)	3.747	0.154
Age (years, x̄±s)	51.5±11.2	58.5±9.6°	66.9±8.1	66.335	<0.001
Han ethnicity [Example (%)]	142 (91.6)	111(91.0)	78 (91.8)	0.050	0.975
Disease course [years, M(Q, Q_3_)]	1.0 (0.3, 4.0)	10.0 (8.0, 11.0)a	15.0 (11.0, 18.0)b	261.049	<0.001
BMI [kg/m^2^ , M(Q_1_ , Q_3_)]	24.00 (22.39, 26.37)	25.10 (23.00, 26.74)	24.40 (22.38, 25.88)	5.502	0.064
SBP (mmHg, x̄±s)	123.75±11.68	130.75±11.36	145.51±17.31	75.541	<0.001
DBP (mmHg, x̄±s)	76.77±8.37	78.23±7.96	82.80±9.85ab	13.700	<0.001
HbA_1_ [%, M(Q_1_, Q_3_)]	8.80 (7.50, 11.70)	8.80 (7.40, 10.52)	9.20 (8.10, 10.80)	2.342	0.310
FPG (mmol/L, MQ_1_, Q_3_)]	8.79 (7.25, 9.86)	9.94 (7.98, 11.86)	9.80 (7.45, 11.91)	15.028	0.001
FC-P [ng/mL, M(Q_1_, Q_3_)]	1.47 (1.04, 1.91)	1.29 (0.91, 1.66)	1.02 (0.66,1.51)ab	27.358	<0.001
HOMA-IR [M (Q_1_, Q_3_)]	2.95 (2.58, 3.53)	2.94 (2.54, 3.54)	2.61 (2.17, 3.28)ab	12.908	0.002
TC (mmol/L, x̄±s)	4.43±0.91	4.25±0.92	4.28±1.18	1.383	0.252
TG [mmol/L, M(Q_1_, Q_3_)]	1.72 (1.30, 2.31)	1.54 (1.17, 2.21)	1.52 (1.10, 2.06)	4.124	0.127
HDL-C [mmol/L, MQ_1_, Q_3_]]	1.10 (0.93, 1.25)	1.14 (0.98, 1.34)	1.18 (1.00, 1.36)	3.818	0.148
LDL-C (mmol/L, x̄±s)	3.03±0.84	2.85±0.76	2.84±1.03	2.059	0.129
Smoking [Example (%)]	15 (9.7)	13 (10.7)	15 (17.6)	3.594	0.166
Drinking alcohol [Example (%)]	11 (7.1)	5 (4.1)	9 (10.6)	3.297	0.192

### Comparison of 25(OH) D and inflammatory indi cators among the three groups

The levels of 25(OH) D in both the T2DM microvascular lesion group and the T2DM macrovascular lesion group were significantly lower than those in the simple T2DM group (P<0.05). The level of IL-6 in the T2DM microvascular lesion group was considerably greater than that in the simple T2DM group (P< 0.05). The levels of CRP IL-6 and the SII in the T2DM macrovascular lesion group were significantly greater than those in the simple T2DM group and the T2DM microvascular lesion group (P<0.05, Table 2).

**Table 2:** Comparison of 5(OH)D and inflammatory indicators in 3 groups of T2DM patients [M (Q-i, Q3)].

Observation index	Simple T2DM group (155 cases)	T2DM microvascular lesion group (122 cases)	T2DM macrovascular lesion group (85 cases)	H value	P value
25(OH) D (ng/mL)	13.10 (10.80, 17.47)	11.56 (9.25, 16.90)	11.30 (9.40, 15.10)	15.310	<0.010
CRP (mg/L)	0.56 (0.50, 1.00)	0.62 (0.50, 1.25)	2.51 (1.50, 4.76)	113.748	<0.010
IL-6 (pg/mL)	1.84 (1.50, 2.15)	2.17 (1.50, 2.65)	5.60 (3.84, 6.86)ab	176.312	<0.010
SII (x10^9^/L)	420.00 (315.17, 512.35)	440.30 (319.36, 522.75)	560.85 (440.50, 781.92)	51.861	<0.010

### Logistic regression analysis of factors influencing vascular lesions in patients with T2DM

Following the correction of BMI, SBF, DBF, HbA1c, FPG, HOMA-IR, TC, TG, LDL-C, age, sex, and disease duration, patients in the T2DM microvascular lesion group, when CRP increased by 1 mg/L, the risk of microvascular lesions in those with T2DM increased by 4.35 times (OR=5.35, 95% CI 1.49-19.13). For every 1 ng/mL increase in 25(OH) D, the risk of microvascular lesions in T2DM patients decreased by 12% (OR=0.88, 95% CI 0.78-0.98). In the T2DM macrovascular lesion group, for every 1 mg/L increase in CRP, the risk of T2DM macrovascular lesions increased by 13.99 times (OR=14.99, 95% CI 2.84-79.13), and for every 1 pg/mL increase in IL-6, the risk of T2DM macrovascular lesions increased by 26.92 times (OR=27.92). For every 1 x 109/L increase in the SII, the risk of macrovascular disease in T2DM patients increased 0.01-fold (OR=1.01, 95% CI 1.00-1.02), and for every 1 ng/ml increase in 25(OH) D, the risk of microvascular lesions in T2DM patients was reduced by 26% (OR=0.74, 95% CI 0.60-0.92) (Table 3 and Table 4).

**Table 3:** Univariate sum of influencing factors of microvascular lesions in patients with T2DM Multivariate logistic regression analysis.

Variable	Univariate logistic regression analysis		Multivariate logistic regression analysis	
	OR value (95%CI)	P value	OR value (95%CI)	P value
Gender	0.97 (0.59~1.60)	0.902	1.29 (0.33~5.07)	0.719
Age	1.07 (1.04~1.10)	<0.001	1.06 (1.00~1.14)	0.061
Disease course	2.21 (1.84~2.66)	<0.001	2.79 (2.06~3.76)	<0.001
BMI	1.02 (0.95~1.11)	0.540	1.06 (0.89~1.28)	0.513
SBP	1.05 (1.03~1.07)	<0.001	1.07 (1.01-1.14)	0.023
DBP	1.02 (0.99~1.05)	0.156	1.01 (0.92-1.11)	0.812
HbA1c	0.89 (0.80~1.00)	0.043	0.97 (0.69-1.38)	0.882
FPG	1.18 (1.08~1.29)	<0.001	1.34 (0.97-1.84)	0.077
FC-P	0.68 (0.47~1.00)	0.048	-	-
HOMA-IR	1.04 (0.78~1.39)	0.805	0.96 (0.36-2.51)	0.926
TC	0.83 (0.65~1.06)	0.127	0.38 (0.07-2.08)	0.262
TG	0.96 (0.77~1.20)	0.735	1.30 (0.78-2.15)	0.309
HDL-C	1.99 (0.80~4.94)	0.137		
LDL-C	0.79 (0.60~1.04)	0.091	1.16 (0.17-7.74)	0.877
Han ethnicity	0.92 (0.40~2.14)	0.853	-	-
Smoking	1.11 (0.51~2.44)	0.789	-	-
Drinking alcohol	0.56 (0.19~1.66)	0.294	-	-
25(OH) D	0.97 (0.93~1.01)	0.135	0.88 (0.78-0.98)	0.015
CRP	1.74 (1.12~2.70)	0.0145	1.35 (1.49-19.13)	0.010
IL-6	1.66 (1.19~2.33)	0.003	1.76 (0.81-3.85)	0.155
SII	1.00 (1.00~1.00)	0.162	1.00 (1.00-1.00)	0.854

**Table 4:** Single factors influencing macrovascular lesions in patients with T2DM and multivariate logistic regression analysis.

Variable	Univariate logistic regression analysis		Multivariate logistic regression analysis	
	OR value (95%CI)	P value	OR value (95%CI)	P value
Gender	0.58 (0.34-1.00)	0.048	0.68 (0.03-13.41)	0.800
Age	1.18 (1.14-1.23)	<0.001	1.14 (0.96-1.34)	0.129
Disease course	3.33 (2.67-4.16)	<0.001	5.92 (3.30-10.61)	<0.001
BMI	0.92 (0.84-1.01)	0.073	1.10 (0.71-1.72)	0.657
SBP	1.13 (1.10-1.16)	<0.001	1.27 (1.08-1.48)	0.003
DBP	1.08 (1.05-1.12)	<0.001	1.07 (0.90-1.26)	0.436
HbA1	0.98 (0.88-1.10)	0.772	1.30 (0.58-2.94)	0.528
FPG	1.17 (1.06-1.29)	0.003	0.65 (0.30-1.43)	0.284
FC-P	0.26 (0.15-0.45)	<0.001	-	-
HOMA-IR	0.55 (0.37-0.82)	0.003	0.46 (0.08-2.70)	0.390
TC	0.85 (0.65-1.11)	0.233	0.05 (0.00-4.80)	0.198
TG	0.81 (0.62-1.07)	0.144	0.97 (0.12-8.18)	0.978
HDL-C	2.49 (0.91-6.78)	0.074		
LDL-C	0.77 (0.56-1.05)	0.096	0.82 (0.01-78.93)	0.934
Han ethnicity	1.02 (0.39-2.66)	0.968	-	-
Smoking	2.00 (0.92-4.32)	0.078	-	-
Drinking alcohol	1.55 (0.62-3.90)	0.352	-	-
25(OH) D	0.90 (0.85-0.96)	0.001	0.74 (0.60-0.92)	0.006
CRP	5.67 (3.52-9.12)	<0.00	14.99 (2.84-79.13)	0.001
IL-6	9.56 (5.64-16.19)	<0.001	27.92 (4.24-183.92)	0.001
SII	1.01 (1.00-1.01)	<0.001	1.01 (1.00-1.02)	0.029

### Spearman correlation analysis of 25(OH) D with inflammatory indicators

Spearman correlation analysis indicated that 25(OH) D exhibited a negative correlation with CRP (r=-0.102, P=0.052), IL-6 (r=-0.115, P=0.028), and the SII (r=-0.141, P=0.007) (P<0.05); CRP demonstrated a positive correlation with IL-6 (r= 0.600, P<0.001) and the SII (r=0.256, P<0.001); and IL-6 was positively correlated with the SII (r=0.307, P<0.001) (P<0.05).

## Discussion

Serum inflammatory markers CRP IL-6, and SII were considerably higher in the T2DM macrovascular lesion group than in the T2DM microvascular lesion group, but serum 25(OH) D levels were significantly lower. The levels of serum inflammatory factors, including CRP IL-6, and the SII, are factors influencing T2DM macrovascular lesions. Only the serum inflammatory factor CRP is an influencing factor of microvascular lesions in T2DM patients, and its OR value is significantly lower than that of macrovascular lesions in T2DM patients.

Hyperglycemia activates inflammatory pathways through multiple mechanisms, including oxidative stress, alterations in protein kinase signalling, and specific microRNAs and epigenetic modifications, which impair the function of pancreatic beta cells and contribute to the progression of insulin resistance. Ultimately, hyperglycemia and inflammation jointly promote the occurrence and development of vascular lesions [9].

CRP is an acute-phase protein that is produced when the body is infected or damaged, and it functions by activating the complement system and enhancing the regulatory effect of phagocytes. CRP is not only a nonspecific inflammatory marker but also directly involved in CVD, such as atherosclerosis itself, and is the most powerful predictor and risk factor for CVD. According to earlier research, macrovascular lesions are linked to the blood CRP level in individuals with type 2 diabetes mellitus (T2DM) [10] [11]. After controlling for confounding variables, the study's findings indicate that the serum CRP level in individuals with type 2 diabetes mellitus (T2DM) influences both microvascular and macrovascular lesions. Additionally, the CRP level is more important and damages macrovascular lesions more severely in patients with T2DM.

Helper T2 cells, fibroblasts, vascular endothelial cells, and mononuclear macrophages are the primary producers of IL-6, a cytokine belonging to the chemokine family [12] [13]. IL-6 may hasten the development of macrovascular lesions in T2DM patients [14]. According to domestic research, the increase is greater than in diabetic patients without macrovascular complications [15]. However, the increase was more pronounced in the T2DM macrovascular lesion group, and the difference between the two groups was statistically significant (P < 0.05). The study's findings showed that the serum IL-6 concentration was significantly elevated in both the T2DM microvascular lesion group and the T2DM macrovascular lesion group. An innovative form of inflammatory indicator is SII. Primarily, it was employed to predict unfavourable clinical outcomes for several types of cancer and inflammatory diseases [16]. The SII has been linked to mortality and prog nosis in individuals with cardiovascular disease, according to recent research [17]. Type 2 diabetes mellitus (T2DM) individuals are at risk for cerebral artery stenosis, and the SII has predictive value for T2DM and intracranial artery stenosis [18]. According to the study's findings, there was no difference between the T2DM microvascular lesion group and the simple T2DM group. However, the SII was considerably higher in the T2DM macrovascular lesion group (P < 0.05). After controlling for confounding variables, the serum SII in T2DM patients was a strong predictor of macrovascular lesions but did not substantially correlate with microvascular lesions.

25(OH) D is a steroid derivative and a fat-solu ble vitamin. It is the main storage form of vitamin D in the body. Prospective cohort studies have shown that higher serum 25(OH) D levels are significantly associated with a reduced risk of cardiovascular outcomes and all-cause mortality in patients with prediabetes and diabetes [19]. The results of this study indicate that the serum 25(OH) D level is significantly decreased in T2DM patients with vascular lesions. It is speculated that this factor may be protective against T2DM vascular lesions and that its protective effect is more significant in the T2DM macrovascular lesion group.

The recognised traditional risk factors for T2DM vascular lesions, such as blood lipids, HbA1c, and HOMA-IR, all showed no statistical significance when analysed by univariate logistic regression, suggesting that blood lipids, HbA1c, and HOMA-IR were not correlated with T2DM vascular lesions. The possible reason is that the average duration of diabetes in the included diabetic patients was 8.0 (2.0, 11.3) years, and they had received standardised and systematic treatment. The results for each inflammatory indicator and 25(OH) D concentration were significantly different, which was considered to be related to the small sample size. The sample size will be increased for future studies.

The mechanisms by which CRP IL-6 and the SII cause macrovascular lesions are as follows: cholesterol crystals, neutrophil extracorporeal traps, hypoxia, and atherosclerotic blood flow can activate the inflamma tory cascade, allowing the precursor of IL-1β to progress to active IL-1β, thereby stimulating the body to produce IL-6. IL-6 further promotes the production of CRP in the liver. Activated IL-1β, IL-6, and CRP interact with each other. It causes vasculitis, vascular endothelial cell dysfunction, and atherosclerosis, eventually leading to macrovascular lesions. 1,25(OH) D3 can induce monocyte differentiation into macrophages and reduce the release of inflammatory cells and chemokines. Therefore, 25(OH) D deficiency may lead to macrovascular lesions. There is a complex network system among various inflammatory factors. This study revealed a positive correlation among various inflammatory indicators, and 25(OH) D was negatively correlated with each of these indicators, suggesting a potential protective role.

Glucagon-like peptide-1 receptor agonists (GLP-1RAs) can intervene in atherosclerosis through multiple inflammatory pathways [20]. Both basic research and clinical research [21] [22] [23] [24], have confirmed that GLP-1RA, represented by elaglutide and semaglutide, can improve the chronic low-grade inflammatory state of patients with type 2 diabetes mellitus (T2DM) by reducing the levels of CRP and IL-6 and inhibiting multiple inflammatory pathways, thereby benefiting from macrovascular lesions in T2DM patients. Studies [25] [26] [27] have shown that higher serum 25(OH) D levels (>75 ng/mL) are significantly linearly correlated with lower all-cause mortality (reduced by 31%) and CVD mortality (reduced by 38%). Thus, vitamin D supplements may serve as a potential means for preventing and treating atherosclerosis in diabetic patients [28] [29] [30].

## Conclusion

Compared with those in patients with T2DM microvascular lesions, CRP and IL-6 and the SII in the serum of patients with T2DM macrovascular lesions are greater, and 25(OH) D is lower. Patients with T2DM and macrovascular lesions may benefit from selecting hypoglycemic drugs with anti-inflammatory effects and monitoring their 25(OH) D levels.

## Dodatak

### Authors' contributions

Qian Zhang and Yuyan Zhu contributed equally to this work and are recognised as co-first authors.

### Conflict of interest statement

All the authors declare that they have no conflict of interest in this work.
